# Characterization of *Listeria monocytogenes* Isolates from Pork Production in Southern Sonora, Mexico: Serotyping, Antimicrobial Resistance, Chitosan Susceptibility, and Pathogenicity in a Chicken Embryo Model

**DOI:** 10.3390/foods14173057

**Published:** 2025-08-29

**Authors:** Alejandro Miguel Figueroa-López, Michel Teresa Gutiérrez-Osuna, Norma Gabriela Rodríguez-Mora, Carlos Eduardo Aragón-López, Roberto Rodríguez-Ramírez, Alma Guadalupe Villa-Lerma, Ernesto Uriel Cantú-Soto

**Affiliations:** 1 Departamento Académico de Ciencias Naturales y Exactas, Unidad Regional Los Mochis, Universidad Autónoma de Occidente, Blvd. Macario Gaxiola y Carretera Internacional, Los Mochis C.P. 81223, Sinaloa, Mexico; alejandro.figueroa@uadeo.mx; 2 Departamento de Biotecnología y Ciencias Alimentarias, Instituto Tecnológico de Sonora, 5 de Febrero 818 sur, Cajeme C.P. 85000, Sonora, Mexico; michel.gutierrez211510@potros.itson.edu.mx (M.T.G.-O.); norma.rodriguezmora@potros.itson.edu.mx (N.G.R.-M.); carlos.aragon139216@potros.itson.edu.mx (C.E.A.-L.); roberto.rodriguez@itson.edu.mx (R.R.-R.); alma.villa@itson.edu.mx (A.G.V.-L.)

**Keywords:** contaminated pork meat, chitosan, pathogenesis

## Abstract

*Listeria monocytogenes* is often found in pork intestines and can contaminate pork production, posing a risk to consumers. This study aimed to characterize 16 *L. monocytogenes* isolates from fresh and packaged pork loin, identify their serotypes, and assess antibiotic resistance. To evaluate chitosan susceptibility as a potential strategy to control *L. monocytogenes* in the pork industry and to determine its effectiveness in a eukaryotic model to demonstrate pathogenicity. Among the 16 isolates examined, 2 were identified as 1/2a, 12 as 1/2b, 2 as 4b, and 2 could not be assigned a serotype. Variations were observed in their pathogenicity factors. Some isolates were lacking in some virulence factors. In the antibiotic assays, all isolates demonstrated resistance to at least three antibiotics, and one of them exhibited resistance to as many as ten antimicrobial agents. To propose an alternative in the food industry as a decontamination agent, a low-molecular-weight chitosan was evaluated. It was shown that chitosan inhibits the growth of *L. monocytogenes* in a concentration of 0.25% in 45 min, resulting in a viable alternative against this pathogen, but in this work, one isolate exhibited resistance to chitosan (isolate Lm 1.2). Regarding infection in eukaryotic models, all isolates had the capacity to infect chicken embryos, except for isolate 1.2, which exhibited attenuated pathogenicity. These findings highlight the potential public health risk *L. monocytogenes* poses in pork and the need for continued research to develop effective control strategies.

## 1. Introduction

*Listeria monocytogenes* is a pathogen found in foods and has shown persistence in food-related environments [1]. *L. monocytogenes* belongs to the genus *Listeria,* comprising 20 other species, some recently described [2]. Only *Listeria ivanovii* and *L. monocytogenes* are pathogenic to humans and ruminants [3,4]. Among these species, *L. monocytogenes* is classified into four genetic lineages, including 14 serotypes. Most food safety authorities worldwide consider all strains of *L. monocytogenes* virulent, although there is significant genetic variability among isolates obtained from diverse sources [5]. Serotypes 1/2a, 1/2b, 1/2c, and 4b are the most commonly responsible for cases of listeriosis, although they may vary in some geographic regions of the world [6,7,8,9].

The primary food sources of *L. monocytogenes* that have been reported include fresh, unpasteurized milk and cheeses, ice cream, fresh or processed vegetables and fruits, fresh or undercooked poultry, sausages, hot dogs, deli meats, and fresh or smoked fish and other seafood. *L. monocytogenes* has also been found in fresh pet food [10]. Ready-to-eat foods represent the primary sources of *L. monocytogenes* contamination, and contaminated fresh meats also represent a risk. Since they are considered a nutrient-rich matrix that supports the growth of microorganisms, *L. monocytogenes* can continue to develop and remain viable even at sub-zero temperatures. This highlights its resilience in food storage environments [11].

*L. monocytogenes*’ ability to persist in the processing environment is widely recognized as the primary cause of food contamination. Furthermore, outbreaks of foodborne listeriosis have been associated with contamination of processing facilities by environmental *L. monocytogenes* [12]. This pathogen in cooling and cutting areas significantly contributes to meat contamination [13].

Although raw meat is not directly associated with outbreaks of this pathogen, it can pose a problem when contaminating other foods through cross-contact, such as raw vegetables and salads [14]. It signifies the primary risk phase for foodborne illnesses during domestic food preparation [14].

Human infection with *L. monocytogenes* can cause a foodborne disease called listeriosis, and this infection is responsible for affecting elderly, immune-compromised individuals, pregnant women, children, and can cause abortions [15]. *L. monocytogenes* infection occurs after ingestion of food contaminated with this pathogen. The infection is facilitated by proteins encoded by a specific gene cluster. Once in the host, *L. monocytogenes* escapes from the phagocytic vacuole and initiates multiplication in the host cell cytoplasm. Then, the motility occurs by the induction of actin polymerization at one pole of the bacterial cell. The last step involves motility within cytoplasmic evaginations of the neighboring cells, where the cycle begins again [16]. This intracellular infection/pathogenicity is mediated by some gene products encoded on a six-gene cluster called LIPI-1 (*Listeria* pathogenicity island), about 9 kb long [17]. This cluster consists of a transcriptional master regulator (*prfA*); two phospholipases known as *plcA* and *plcB*, which are involved in lysing host cell membranes; a hemolysin (Listeriolysin-*hlyA*) that is necessary for disrupting the host’s phagosome to access the cytoplasm; a metalloprotease that activates the inactive propeptide *plcB* in the extracellular environment; and the *actA* gene that encodes the surface protein ActA, which is essential for bacterial motility [18,19]. All these genes and their products are involved in the *L. monocytogenes* pathogenicity.

When listeriosis occurs, the main set of antibiotics used to treat this illness include trimethoprim with sulfamethoxazole, ampicillin, tetracycline, gentamicin, erythromycin, penicillin, rifampicin, and chloramphenicol [20]. The indiscriminate use of these compounds can force wild-type isolates to develop multiple antibiotic resistance, and its resistance can range from 3 to 13 antibiotics [20]. Listeriosis is a disease that poses a high risk of death among vulnerable individuals. Out of 1600 people who become ill, 260 die each year (CDC, 2020). The European Union reports a 15.6% fatality rate among reported cases of listeriosis [21].

To minimize the damage caused by *L. monocytogenes*, various control methods have been developed in the meat processing sector within the industry [22]. It is known that *L. monocytogenes* develops tolerance to some bactericides such as benzalkonium chloride, alkylbenzyl-dimethyl ammonium chloride, n-alkyldimethylethylbenzyl ammonium chloride, tertiary alkyl-amine, 1,3-propanediamine-N-(3-aminopropyl) N-dodecyl, sodium hypochlorite, and potassium persulphate [22,23,24]. For this reason, alternative methods have been proposed for its control, such as the use of biopolymers with bactericidal effects [25]. Chitosan, a natural and entirely safe biopolymer, constitutes an exemplary antimicrobial agent within the food industry. Its mechanism of action, which disrupts microbial membranes, facilitates effective control of pathogens and spoilage microorganisms. As a biodegradable and non-toxic substance, it provides a safe and sustainable alternative to chemical preservatives. Its application in coatings or additives prolongs food shelf life and enhances safety without compromising consumer health. In this sense, chitosan is a biopolymer derived from shrimp waste that has been tested as an alternative for the potential development of a product against *L. monocytogenes* [25].

The aims of this study were to characterize sixteen *L. monocytogenes* isolates from fresh pork loin and fresh packaged pork loin, identify their serotypes and antibiotic resistance profiles, assess their susceptibility to chitosan as a possible strategy to control *L. monocytogenes*. In studies related to the characterization of *Listeria monocytogenes*, its pathogenic potential is rarely or never evaluated in a eukaryotic model. In this work, we wanted to address this issue to find out whether all *Listeria monocytogenes* clones are pathogenic or whether there is any variation in pathogenic behavior in a eukaryotic model.

## 2. Materials and Methods

### 2.1. Sampling

Four sampling events, each lasting two months, were conducted at a regulated slaughterhouse south of Sonora, México. Ten samples of raw pork loin were collected in the first sampling event. Six samples of raw pork loin were collected in the second sampling event. These samples of fresh pork loin were collected in sterile bags. In the third sampling event, six samples of fresh packaged pork loin were collected. In the fourth sampling event, four samples were taken. Samples of fresh packaged pork loin were collected in their final packaging. Samples were preserved at a low temperature of 4–8 °C and analyzed on the day of collection.

### 2.2. Isolation and Phenotypic Characterization of Listeria monocytogenes

All isolates were obtained from fresh pork loin and packaged fresh pork loin, following the procedure described in Appendix C of the Mexican Standard NOM 210-SSA-2014, which resembles the method outlined by the International Organization for Standardization in ISO 11290 for *L. monocytogenes*. Briefly, 25 g of the sample was mixed with 25 mL of phosphate-buffered saline (137 mM NaCl, 10 mM phosphate, 2.7 mM KCl, and pH 7.4) in a sterile blender in aseptic conditions. Then, each 25 mL from each sample was added to 225 mL of Fraser Broth (Merck, Cat. 63017-500, Darmstadt, Germany) containing Fraser supplements (Sigma-Aldrich, Cat. 90836-10VL; Darmstadt, Germany), and incubated for 48 h at 36 °C. Then the sample was plated on Oxford plates (Condalab, Cat. 1133; Torrejón de Ardoz, Madrid, Spain) with supplements (Oxoid, Cat. SR0140E; Waltham, MA, USA) and incubated for 48 h at 36 °C. The round grayish colonies surrounded by dark halos were used in the following tests. The biochemical characterization was carried out according to Momtaz and Yadollahi: Gram staining; catalase and oxidase tests, motility test at 25 °C, acid production from rhamnose and xylose, the β-hemolytic activity on sheep blood agar test, and the CAMP test (Christie, Atkins, Munch-Peterson test) [26]. *Staphylococcus aureus* ATCC 29213 (SA+) and *Rhodococcus equi* ATCC 6939 (RE−) were used as controls for the CAMP test. *Listeria monocytogenes* ATCC 7644 was used as the positive control for all tests. All positive isolates to *L. monocytogenes* were recovered and stored at −70 °C with glycerol (15%) until used.

### 2.3. Genotypic Characterization of Listeria monocytogenes Isolates

#### 2.3.1. DNA Extraction

A colony from a previous culture for each isolate was used to inoculate 5 mL of Trypticase Soy Broth (Cat. No. 211768; BD Biosciences, Franklin Lakes, NJ, USA) and then incubated at 37 °C for 24 h. Subsequently, the cells were harvested by centrifugation at 5000× *g* for 3 min, and the cell pellet was used for DNA extraction. Briefly, the cell pellet of each isolate was resuspended in a lysis buffer (0.03 M Tris-HCl, 0.01 M EDTA, and 20 g/L lysozyme [Sigma-Aldrich Lysozyme from chicken egg white. Cat. No. 6876; Burlington, MA, USA]) and incubated at 37 °C for 30 min. Genomic DNA was extracted with the Qiagen^®^ DNeasy^®^ Blood and Tissue Kit (Qiagen Cat. No. 69504; Hilden, Germany) according to the manufacturer’s instructions. The DNA concentration and purity were measured using a Nanodrop 2000c UV–Vis spectrophotometer (ThermoFisher, Inc.; Wilmington, DE, USA).

#### 2.3.2. Detection of Virulence Factors by PCR

The detection of virulence factors was performed using the polymerase chain reaction. For amplifying the virulence factors *hlyA*, *iap*, *actA*, *prfA*, and *plcA*, the oligonucleotides reported by Momtaz et al. [26] were used. For amplifying virulence factors *plcB*, *InlA*, *InlB*, *InlC*, and *InlJ*, we designed specific oligonucleotides using the Primer3 plus program. The components of the reaction were the following: 1× PCR buffer, 2 mM of MgCl_2_, 0.2 μM of dNTPs, 0.2 μM of each oligonucleotide, 1 U of Taq DNA polymerase (Invitrogen™. Cat. No. 18038042; Waltham, MA, USA), 10 ng of DNA and water until 25 uL. All the primers used are listed in Table S1. A Labnet, MultiGene optiMAX machine (Edison, NJ, USA) was used to run the reactions. The conditions used were an initial denaturation step at 95 °C for 5 min, 30 cycles at 95 °C for 30 s, annealing for 30 s and the melting temperature is indicated in Table S1 for each primer pair, and extension at 72 °C for 30 s, and a last step at 72 °C for 10 min.

#### 2.3.3. Molecular Serotyping of Wild Isolates of *Listeria monocytogenes*

All isolates recovered were serotyped by PCR, as described by Doumith et al. [6]. The set of primers used for serotyping are listed in Table S1. These primer pairs target the three major serovars of *L. monocytogenes*. The set of lmo0737 amplifies a region of 691 bp of the putative protein gene with an unknown function to detect the serotype 1/2a. The pair ORF2819 amplifies a region of the putative transcriptional regulator generating a 471 bp fragment to detect the serotype 1/2b. For serotype 4b, the set of primers ORF2110 was utilized to detect a fragment of the putative secreted protein, yielding an amplicon of 597 bp. The components of the reaction were the following: 1× PCR buffer, 2.5 mM of MgCl_2_, 0.8 mM of dNTPs, 0.4 μM of each oligonucleotide, 1 U of Taq DNA polymerase (Invitrogen™. Cat. No. 18038042; Waltham, MA, USA), 10 ng of DNA and water until 12.5 uL. The PCR conditions included an initial denaturation step at 95 °C for 7 min, followed by 30 cycles at 95 °C for 15 s, annealing at 56 °C for 20 s, and extension at 72 °C for 60 s, and a last step at 72 °C for 10 min. PCR products were loaded and electrophoresed in a 1% agarose gel in 0.5 X Trisacetate EDTA buffer, stained with ethidium bromide, and visualized on a Minibis Pro DNR system (Bio-Imaging systems).

### 2.4. Assessment of the Antibiotic Resistance Profiles of Wild-Type Listeria monocytogenes Isolates

To confirm susceptibility/resistance to antibiotics an analysis of minimum inhibitory concentration (MIC, μg/mL) was analyzed on 68 Microscan GN systems according to the Clinical and Laboratory Standard Institute (CLSI) [27]. The inhibition was evaluated based on the breakpoints criteria established in the CLSI guidelines for *Staphylococcus aureus* because no resistance criteria exist for *Listeria monocytogenes* susceptibility testing in the CLSI guidelines. Twenty-one antibiotics were tested against all isolates: AMC, amoxicillin-clavulanic acid; SAM, ampicillin-sulbactam; AMP, ampicillin; CRO, ceftriaxone; CIP, ciprofloxacin; CLI, clindamycin; DAP, daptomycin; ERY, erythromycin; FOF, fosfomycin; GEN, gentamicin; LVX, levofloxacin; LZD, linezolid; MXF, moxifloxacin; NIT, nitrofurantoin; OXA, oxacillin; PEN, penicillin; RIF, rifampin; SYN, synercid; TET, tetracycline; SXT, trimethoprim-sulfamethoxazole; and VAN, vancomycin. The multiple antibiotic resistance (MAR) index was calculated according to Singh et al. [28]. MAR index MARI = number of resistance antibiotics/total number of antibiotics tested. The strains that resisted at least three antibiotic compounds (>0.143) were considered multiple antibiotic resistance (MAR).

### 2.5. Antimicrobial Assays with Shrimp Chitosan Against Listeria monocytogenes Isolates

#### 2.5.1. Chitosan Preparation

Chitosan was obtained following the process of Rodríguez-Núñez et al. [29]. Chitin extraction was carried out on a pilot scale using lactic fermentation, following the procedure outlined by Bueno-Solano et al. [30]. To obtain chitosan with a low molecular weight, the method described by Weska et al. [31] was employed with certain modifications. Initially, the chitin was subjected to a protein removal process by immersing it in a 4.5% (*wt*/*vol*) NaOH solution at 65 °C for 4 h. Subsequently, the resulting solid precipitate underwent a mineral removal step by immersion in a 3.6% (*wt*/*wt*) HCl solution at room temperature for 4 h. The final stage involved an alkaline deacetylation process using a 45% (*wt*/*vol*) NaOH solution at 120 °C for 2 h, followed by thorough rinsing with water and drying at 40 °C for 12 h. Prior to utilization, the chitosan was finely powdered through milling to achieve a particle size of 180 μm.

#### 2.5.2. Chitosan Antimicrobial Assay

To establish the chitosan concentration and exposure time to inhibit the *L. monocytogenes* growth, a preliminary experiment was conducted using three concentrations and three exposure times. The concentrations of 0.1, 0.175, and 0.25% (*wt*/*vol*) diluted in 1% acetic acid were evaluated at 7.5, 26, and 45 min exposure times. The treatments were incubated at 37 °C for 45 min, harvesting at 7.5, 26, and 45 min and serial dilutions were made to count the CFU/mL for each treatment. Once the appropriate chitosan concentration (0.25% *wt*/*vol*) and time (45 min) were selected and evaluated with the wildtype strains of *L. monocytogenes* (Figure S1). To discard the effect of acetic acid on bacterial development and only attribute the antimicrobial effect to chitosan, a treatment with only acetic acid was evaluated (1% *vol*/*vol*). Before carrying out the test, each strain was thawed and grown in 5 mL of Trypticase Soy Broth (Cat. No. 211768; BD Biosciences, Franklin Lakes, NJ, USA) for 24 h at 37 °C. After that, 1 mL was taken and inoculated in a flask with 99 mL of Trypticase Soy Broth (Cat. No. 211768; BD Biosciences, Franklin Lakes, NJ, USA), shaken, and incubated for 7 h at 37 °C. Subsequently, 1 μL of the 7 h culture (approximately 1 × 10^6^ cells/μL) was taken and inoculated into a 1.5 mL tube containing 999 μL of each treatment. The cell concentrations were expressed in Log of CFU/mL. At the end of the incubation with each treatment, the cells were recovered and subsequently plated onto Trypticase Soy agar plates in order to enumerate the viable cells. The results of the counts were converted to base 10 logarithms. A Shapiro–Wilk test was used to analyze the normality of the data. A one-way ANOVA test was performed to determine whether there were variations between treatments (*p* < 0.05). Variations between groups were identified using a Tukey HSD test at 95% confidence. The analyses were performed using STATGRAPHICS Plus 5.0 software.

### 2.6. Assessing the Virulence of Listeria monocytogenes Isolates on Chicken Embryos

#### 2.6.1. Checking for the Dead Embryos

This assay was carried out according to Andersson et al. [32] with modifications. Briefly, this experiment was performed in chicken embryos (*Gallus gallus*); the eggs were incubated at 37.5 °C with 65% relative moisture for 8–9 days to discard dead embryos.

#### 2.6.2. *Listeria monocytogenes* Inoculum

All isolates from this study, including the bacterial controls (*Listeria monocytogenes* ATCC 15313 and *Listeria monocytogenes* ATCC 7644), were plated on Brain Heart Infusion Agar (Cat. No. 237500; BD Biosciences, Franklin Lakes, NJ, USA) for activation. A sample was taken from each bacterial vial stored at −70 °C, and the sample was then plated and incubated at 30 °C for 24 h. A single colony was used to inoculate a tube containing 5 mL of Brain Heart Infusion Broth (BIHB) (Cat. No. 237500; BD Biosciences, Franklin Lakes, NJ, USA) and incubated at 30 °C for 24 h. After incubation, 1 mL was taken to inoculate a flask containing 50 mL of BIHB and incubated for 24 h at 30 °C. From this flask, 1 mL is transferred to a 1.5 mL Eppendorf tube and centrifuged at 4000× *g* for 10 min to collect the bacterial cells, which are then resuspended in 1 mL of 0.9% (*wt*/*vol*) saline solution (NaCl). The bacterial cell concentration was adjusted and used to infect the chicken embryos.

#### 2.6.3. Infecting the Chicken Embryos with *L. monocytogenes* Wild Type Isolates

All the viable embryos selected were injected with 100 µL containing 5 × 10^5^ cell forming units (CFU). The eggs were perforated carefully with forceps. The infective dose was injected with a Syringe (0.5 mL, BD Plastipak, Cat. No. U100, East Rutherford, NJ, United States.). Paraffin was applied to the opened egg with cotton, and a small piece of tape was used to cover the opening. The eggs were returned to the incubator. The embryo’s viability was measured every 24 h until the end of the experiment (120 h). The embryos were inspected in a dimly lit area using a lamp. An ovoscopy was performed using an ovoscope on the side opposite the air chamber. Embryos exhibiting blood vessel formation and movement were regarded as alive. Embryos exhibiting fragmented blood vessels and immobility were deemed lifeless. The experiment was carried out three times with five replicates per treatment. Mortality was calculated as the percentage of the total of embryos inoculated for each bacterial treatment during 5-day incubation. A solution of buffered saline was used as a negative control. *Listeria monocytogenes* ATCC 15313 and *Listeria innocua* ATCC 33091 were used as negative controls in the chicken embryo infection because they lack the ability to infect. *Listeria monocytogenes* ATCC 7644 was used as a positive control for embryo infection. This experiment was carried out in an egg incubator at 37 °C and a moisture level of about 65%.

#### 2.6.4. *L. monocytogenes* Detection in Chicken Liver Embryos

To verify whether *L. monocytogenes* had caused an infection, PCR detection was performed in the liver of the chicken embryo. Once the embryos were dead, they were removed from the incubator and analyzed. Each egg was opened on a sterile Petri dish. The embryo was separated from the fluids with forceps and washed with saline solution (0.9% (*w*/*v*) NaCl). The liver was extracted following the instructions of Andersson et al. [32]. The liver was placed in a sterile 1.5 mL Eppendorf tube containing 1 mL of saline solution (0.9% (*wt*/*vol*) NaCl). Total DNA was extracted with Wizard^®^ Genomic DNA Purification Kit (Cat. No. A1125; Promega, Madison, WI, USA) following the manufacturer’s instructions for cultured cells and animal tissue. The DNA was quantified using a Nanodrop 2000c UV–Vis spectrophotometer (ThermoFisher, Inc.; Wilmington, DE, USA). The *L. monocytogenes* detection was carried out for the *hly*A gene as previously described in Section 2.3.2 Detection of virulence factors by PCR.

#### 2.6.5. Ethical Declaration for the Use of Chicken Embryos

This study was conducted with the approval of the Ethics Committee for Research and Animal Welfare of the Instituto Tecnológico de Sonora (Institutional Approval Number was 2024-09). All necessary precautions were taken to ensure the welfare of the chicken embryos used, in compliance with international and national regulations for the use of animals in scientific research.

## 3. Results

### 3.1. Identification of Listeria monocytogenes Isolates

*Listeria monocytogenes* was detected in 9 out of 16 samples of raw pork loin (Table 1). In raw packaged pork loin, the prevalence was 70%, resulting in seven contaminated samples. A total of 16 isolates were obtained from raw and packaged pork loin (Table 1).

### 3.2. Prevalence of Listeria monocytogenes in Fresh Pork Loin and Fresh Packaged Pork Loin

The prevalence is not the central focus of this work, but it is worth mentioning. Sixteen samples out of twenty-six from fresh pork loin and fresh packaged pork loin were found contaminated with *L. monocytogenes*, giving 61.5% of the total prevalence (Table 1). In this case, fresh packaged pork loin samples were highly prevalent (70%), with seven contaminated samples giving seven confirmed isolates (Table 1). Of sixteen fresh pork loin samples, nine were contaminated, and a prevalence of 56.3% was observed, leading to the isolation of nine *L. monocytogenes* strains (Table 1).

### 3.3. Serotype Identification of Listeria monocytogenes Isolates

This study focused solely on serotypes 1/2a, 1/2b, and 4b, previously reported as the most frequent in human listeriosis cases [33]. Here, serotype 1/2b was the most predominant, with 68.7% in both types of samples (Table 1). Two isolates were classified as 1/2a, and one isolate was classified as serotype 4b (Table 1). Two isolates could not be classified using the employed method (Table 1). These isolates will require additional studies to determine their specific serotype.

### 3.4. Detection of Virulence Factors in Listeria monocytogenes Isolates

The *L. monocytogenes* isolates showed different virulence genes presence; most isolates possess all genes evaluated (Table 2). Some of them lack genes; isolates 3.1 and 3.2 lack *actA* and *plcA* genes, and isolate 1.2 lacks *InlA*, *InlB*, and *InlJ*. The *plcA* gene is not present in isolate 7.2, and the *InlB* is not present in isolate 10.1 (Table 2).

### 3.5. Antibiotic Resistance of Wild-Type Listeria monocytogenes Isolates

The main concern regarding foodborne pathogens is antibiotic resistance. In cases of listeriosis, penicillin, ampicillin, and gentamicin are the primary antibiotics employed for treatment [34]. *L. monocytogenes* wild-type isolates showed 100% susceptibility to amoxicillin-clavulanic acid; ampicillin-sulbactam, ampicillin, erythromycin, fosfomycin, gentamicin, moxifloxacin, rifampin, trimethoprim-sulfamethoxazole and vancomycin (Table 3). Here, 100% of isolates were resistant to ceftriaxone, clindamycin, daptomycin, and oxacillin, and 93.3% were resistant to nitrofurantoin. Fifty-three point three percent of the isolates showed resistance to ciprofloxacin. Moreover, 20% of the isolates resisted levofloxacin, linezolid, and synercid (quinupristin/dalfopristin) (Table 3). In this research, 13.3% of the isolates resisted penicillin, while only 6.6% resisted tetracycline (Table 3). *L. monocytogenes* has developed resistance to various antimicrobial compounds. Isolate 1.2 showed the highest Multiple Antibiotic Resistance Index (MARI) at 0.476, followed by isolate 2.1 at 0.429, and isolate z44 at 0.381. The lowest value shown for the MAR index was 0.238, which was present in most isolates (Table 3). An important finding of this study is that a bacterial isolate was found to possess resistance to up to 10 antibiotics (Isolate 1.2). These isolates may be representing a risk for people who may be in contact with them.

### 3.6. Chitosan as an Alternative to Use Against Listeria monocytogenes in the Food Industry

The chitosan used here had a molecular weight of 114.83 kDa (low molecular weight chitosan), viscosity of 427.83 cp, deacetylation’s degree of 92.33% and density of 868.34 kg/m^3^. As reported in other works, a 0.25% of chitosan was used and 45 min exposition for elimination of *L. monocytogenes* (Figure S1). A concentration of 0.25% at 45 min could inhibit the growth of most isolates. Particularly, the isolate 10.1 was not affected by the polymer, this isolate was evaluated six times to avoid mistakes, and the results were always consistent showing resistance against chitosan (Table 4). Acetic acid did not impact the growth of *L. monocytogenes*, therefore the observed inhibition is associated with the chitosan used (Table 4).

### 3.7. Listeria monocytogenes Infection Assays on the Chicken Embryo Model

All isolates (Lm 1., Lm 1.2, Lm 1.3, Lm 7.1, Lm 7.2, Lm 7.3, Lm Z44, Lm 10.1, Lm 10.2, Lm 2.1, Lm 2.3, Lm 3.1, Lm 3.2, Lm 3.3, Lm 42.1, Lm 42.2) were evaluated in chicken embryos to examine whether they could cause an infection.

**Figure 1:**
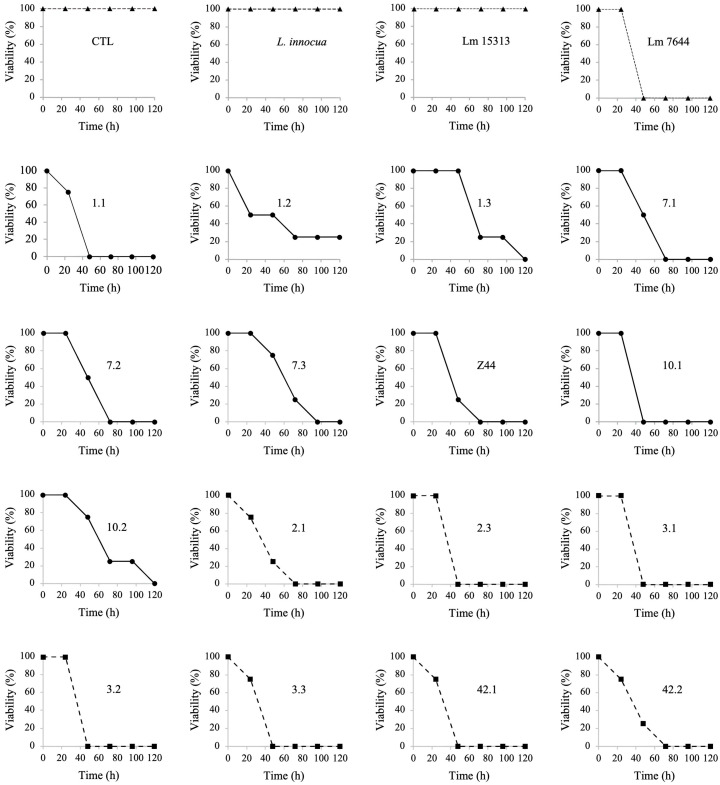
Mortality rates in chicken embryos post-inoculation with wild-type *Listeria monocytogenes* isolates over 120 h. CTL, control solution with NaCl 0.9%. Lm 15313, negative infection control *Listeria monocytogenes* ATCC 15313. *L. innocua*, negative infection control *Listeria innocua* ATCC 33091. Lm 7644, positive infection control *Listeria monocytogenes* ATCC 7644. Graphs with filled triangles and dotted lines belong to the control strains. Graphs with filled circles belong to isolates from raw pork loin. Graphs with filled squares and dotted lines belong to isolates from raw package pork loin.

The negative controls CTL (NaCl 0.9%), *L. monocytogenes* ATCC 15313, and *L. innocua* ATCC 33091 did not affect the viability of chicken embryos (Figure 1). *Listeria monocytogenes* ATCC 7644 triggered the mortality of chicken embryos 48 h after infection.

In this experiment, we highlight that the isolates from fresh packaged pork loin were more aggressive than those obtained from fresh pork loin, reaching 100% mortality 48 h post-infection. Five (2.3, 3.1, 3.2, 3.3, and 42.1) of the seven isolates from fresh packaged pork loin showed 100% mortality at 48 h post-infection, and the remaining two (2.1 and 42.2) reached 100% at 72 h post-infection (Figure 1). In fresh pork loin, two isolates (1.1 and 10.1) showed 100% mortality 48 h after infection. Three isolates (7.1, 7.2, z44) showed 100% mortality up to 72 h, one (7.3) at 96 h, and two (1.3 and 10.2) at 120 h. One isolate (1.2) showed only 80% mortality, leaving 20% viable embryos (Figure 1). To confirm the infection in a chicken embryo, DNA was extracted from the livers of infected embryos. The *hylA* gene was used to detect *L. monocytogenes* by PCR. In embryos treated with saline solution (NaCl 0.9%), *L. innocua* ATCC 33091, and *L. monocytogenes* ATCC 15313, the *hlyA* gene was not detected (Table S2).

## 4. Discussions

### 4.1. Identification of Listeria monocytogenes in Raw and Packaged Pork Samples

All isolates obtained showed biochemical behavior as *L. monocytogenes* and were confirmed by PCR using the *hlyA* gene, a common gene used for *L. monocytogenes* confirmation [35]. This finding confirms the presence of the LLO-encoding gene, a crucial toxin associated with the pathogenicity of *L. monocytogenes*, which indicates the potential virulence of these isolates [35]. Indeed, the *hlyA* gene plays a crucial role in identifying *L. monocytogenes*, making it the most frequently selected target among other virulence genes for PCR detection of this pathogen. Molecular detection is less time-consuming than biochemical methods, increasing accuracy and effectiveness [35].

### 4.2. Prevalence of Listeria monocytogenes in Raw and Packaged Pork Samples

Food safety is a significant global concern. Contamination of food, particularly by *L. monocytogenes*, can result in serious consequences. *L. monocytogenes* contamination not only causes economic losses for the food industry but also poses severe health risks, such as miscarriage and food poisoning. Given the persistent nature of this pathogen, it is essential for the food industry to implement regular cleaning and disinfection practices to ensure safety [36]. In Colombian swine, the prevalence of *L. monocytogenes* in fresh pork meat was reported to be 33.9%, less than this work [37]. In other work about raw pork meat from processing plants, the *L. monocytogenes* is similar to this work, with a 37% prevalence reported [38]. Sixteen isolates were obtained from both fresh pork loin and fresh packaged pork loin, confirming the presence of *L. monocytogenes* in fresh pork meat. The contamination of *L. monocytogenes* can occur at various stages of the pork production chain, primarily due to inadequate cleaning and disinfection processes. This allows the pathogen to become established in production equipment [13]. These findings of *L. monocytogenes* levels indicate that the current sanitizing techniques and cleaning agents are inadequate for eliminating this pathogen, emphasizing the need for a more thorough sanitization process.

### 4.3. Serotypes of Listeria monocytogenes in Raw and Packaged Pork Samples

The incidence of listeriosis has been reported to range between 1 and 10 cases per million population annually, with higher rates observed in certain countries [33]. Listeriosis is particularly severe in individuals with weakened immune systems, pregnant women, and newborns, with an estimated mortality rate of 20–30% among clinical cases. To prevent listeriosis, efforts should focus on controlling food production and handling, as well as educating consumers, especially those in high-risk groups, about the risks of exposure and the precautions they should take. Research has examined the prevalence of *L. monocytogenes* serotypes in pork loins and surfaces throughout the production chain, revealing the presence of serotypes 1/2a (19%) and 1/2b (80.95%) [36]. In pork meat, the predominant serotypes are typically 1/2a and 1/2c, followed by 1/2b [39]. Although serotype 4b has also been isolated from pork products [40], it is not the most commonly occurring serotype. One notable study found that the serotypes in pork loins, 1/2a, 1/2b, and 4b, are present in the chain meat processing [41]. *L. monocytogenes* serotypes in pork loins are a significant concern throughout the production chain. Serotypes 1/2a, 1/2b, and 4b have been identified, emphasizing the need for targeted interventions to address specific strains of concern. Ongoing surveillance, continuous improvement of food safety practices, and consumer awareness are essential to reduce the burden of listeriosis associated with pork products.

### 4.4. Virulence Factors Present in Listeria monocytogenes Isolates from Raw and Packaged Pork Samples

The most common virulence factors were detected by PCR to determine the virulence profile associated with *L. monocytogenes* pathogenicity. Other studies had reported similar findings, documenting variability in the virulence genes detected [42,43].

The products of the virulence genes are necessary for *L. monocytogenes* infection, *prfA* is the master regulator of most of these genes. The phospholipases (*plcA* and *plcB*) are required to lyse the host cell membrane and to escape from host cell’s phagosomes *L. monocytogenes* expresses the pore-forming sulfhydryl-activate listeriolysin (LLO product of the gene *hlyA*). The *actA* gene produces an actin assembly surface protein that is responsible for the movement within host cells [17]. The internalins (*lnlA*, *InlB*, *InlC*, *InlJ*) are proteins that are found in bacterial membrane involved in the invasion and adhesion to different types of eukaryotic cells [44].

The *iap* gene is a virulence factor needed for the invasion of *L. monocytogenes* into host cell, and possesses a murein hydrolase activity involved in bacterial cell division [45]. Most wild *L. monocytogenes* isolates evaluated in this work possess all the genes necessary for proper infection of the eukaryotic cell. The absence of the gene *actA* had been reported previously in isolates from meat products in Poland by Kawacka et al. [46], where some isolates showed a rate of 10% to 78% of *actA* presence. Similar results were obtained in Romania from ready to eat products, the *actA* gene was not detected in isolates from samples of years 2019 and 2020 [43]. The internalins in some isolates are absent, a study on genome analysis showed differences in the presence of internalin genes.

Here, the authors mention that *L. monocytogenes* can have different virulence profiles, including the internalin genes [5]. In this work, the gene *plcA* was absent in three isolates, by Coroneo et al. [42], where the *plcA* gene was less predominant in 20% of the isolates evaluated. Here, highlight some genotypic variations according to the presence or absence of virulence factors. This study demonstrates that, within a single species, there are variations in the virulence factors possessed by these bacteria.

The absence of specific genes could indicate a reduction in virulence; however, in this case, the only isolate that showed reduced virulence was *L. monocytogenes* 1.2, as indicated by the results in chicken embryos (shown below). The reduction in its virulence could be attributed to the lack of *InlA*, *InlB*, and *InlJ* genes. Most of the isolates obtained in this study (11 isolates: 7 (1/2b), 2 (1/2a), and 2 ND)) possess genes that are involved in the production of virulence proteins necessary for causing infections in eukaryotic organisms. This presents a potential risk for individuals who may come into contact with contaminated meat. Additionally, understanding the presence of these genes could be beneficial for several purposes: developing vaccines against these bacteria, creating new antibacterial drugs that target these genes, and implementing strategies for timely detection in food and clinical samples. This information may also aid in the quicker and more accurate identification of listeriosis outbreaks.

### 4.5. Antibiotic Resistance of Wild-Type Listeria monocytogenes Isolates from Raw and Packaged Pork Samples

In bacteria, evolutionary mechanisms can develop that allow them to resist the harmful effects of antibiotics. Among these mechanisms are three very common ones that confer resistance to several groups of antibiotics; although specific for each type of compound, their function is similar: (i) efflux pumps, (ii) modifications in the ribosome binding sites, and (iii) enzymatic inactivation of the antibiotic compound. For tetracycline [47], lincosamides [48], oxazolidinones [49], and streptogramins [50], resistance mechanisms like efflux pumps, ribosomal modifications, and enzymatic inactivation have been described. For β-lactam antibiotics, bacteria can develop another mechanism like membrane modification and changing the cell wall structure [51]. For lipopeptides like daptomycin, microorganisms modified some regulatory networks, cell wall structures, and efflux pumps [52]. For fluoroquinolones, although efflux pumps and inactivation enzymes, bacteria require other modifications that confer resistance, like mutations in the topoisomerase gene [51]. For nitrofurantoin, a specific mutation is required, this is focused on nitroreductase genes avoiding the nitrofurantoin activation [53]. The resistance of *Listeria monocytogenes* to β-lactam [54], tetracycline [55], lincosamides [56], fluoroquinolones [57], oxazolidinones [56], lipopeptides (daptomycin) [52], streptogramins (Synercid) [58], and nitrofurantoin [59] were previously documented.

A reference parameter recently used to assess antibiotic resistance is the MAR Index; in this study, all strains recorded values >0.143 were considered multiple antibiotic-resistant strains. This resistance might have originated due to prolonged exposure to sublethal concentrations of antimicrobial compounds and sanitizers used in food processing facilities, which create stressful conditions and foster the development of antibiotic resistance [60]. MAR isolates might also arise from the indiscriminate use of antimicrobials in pork production. Furthermore, if these MAR isolates contaminate the food processing chain, they can be spread to humans through contaminated food and potentially cause a disease [61].

The emergence of multi-resistant bacteria is a problem of global concern. The extensive use of antibiotics exerts a selective pressure that allows bacteria with resistance genes to survive and multiply. Antibiotic resistance can also arise spontaneously due to chromosomal mutations. These mutations serve as a starting point for the development of antibiotic resistance [62,63]. It is well established that bacteria can exchange genetic elements, including genes, integrons, transposons, and plasmids, which may confer resistance to antimicrobial agents. There are two ways in which these elements can be transmitted. First, they can pass from one generation to the next through simple vertical bacterial division. Second, they can act as donors and transfer these elements horizontally to other strains of the same species or genus [64]. The excessive use of antimicrobials, even in animals, promotes the development and dissemination of resistant strains, posing a worldwide threat to public health [65,66].

The impact on human health is significant, particularly due to the lack of effective antibiotics, which can increase mortality rates. Infections will be more difficult to treat, leading to higher economic costs due to prolonged illnesses, which may burden hospitals. These infections could cause a loss of economic productivity in society, with greater effects in developing countries or countries with fewer economic resources. Additionally, these bacteria would heighten the risks associated with medical procedures such as transplants, chemotherapy, and major surgeries because these procedures rely on effective antibiotics to prevent infections. This emergence of multidrug-resistant bacteria in recent years is attributed to the overuse of [67] antimicrobials in humans, agriculture, and animal husbandry. There is a need to raise awareness in the population about the responsible use of antibiotics and to implement monitoring systems to detect the spread of multidrug-resistant bacteria.

### 4.6. Chitosan’s Antimicrobial Effectiveness Tested Against Listeria monocytogenes

Chitosan is a biodegradable and non-toxic biopolymer with antimicrobial and antioxidant properties. There has been an increase in chitosan applications such as wastewater treatment, agriculture, biomedicine, pharmaceuticals, cosmetics, and the food industry, generating a positive impact on the environment by contributing to the reduction in pollution by replacing the indiscriminate use of chemicals [68]. Chitosan is a polysaccharide extracted from chitin that can exert antimicrobial activity against a broad range of foodborne pathogens, and its function depends on the source of chitosan and factors like molecular weight, particle size, pH, temperature, salinity, divalent cations, chitosan solvent, and suspended medium [69].

These chitosan compounds had low molecular weights and were obtained from shrimp shells, as the chitosan used in this study [70,71]. It has been reported that concentrations of 0.02 to 0.5% of chitosan can inhibit *L. monocytogenes* [70,71]. In this study, a concentration of 0.25% applied for 45 min successfully inhibited *L. monocytogenes*, decreasing its counts from 6 to 0 Log CFU/mL. Additionally, concentrations of 0.15% have also proven effective against both *L. monocytogenes* and *Salmonella Typhimurium* [72]. Regarding the acetic acid effect, in this work, the concentrations of acetic acid had no effect on *L. monocytogenes* Log CFU/mL, contrasting with previous work by Ibañez-Peinado et al. [72], where the acetic acid had a reducing effect on the initial Log CFU/mL used in their experiments, and their findings revealed that higher pH values of the chitosan solutions are less effective than low pH values. Most studies on chitosan against foodborne pathogens address its antimicrobial effect, but the mechanisms by which bacteria develop resistance to this polymer remain unclear [73]. A study carried out in *S. aureus,* a Gram-positive bacterium, documented that modifications to cell surface properties can reduce the negative charge of the cell wall and cell membrane, causing a reduced chitosan binding; this suggests that modifications of the cell wall and cell membranes confer resistance to chitosan [73]. Possibly, *L. monocytogenes* developed a similar mechanism explaining the resistance to chitosan observed in this study.

The utilization of chitosan within the food industry is increasingly attracting considerable attention, particularly subsequent to the acknowledgment by the U.S. Food and Drug Administration that chitosan obtained from shrimp is Classified as Generally Recognized as Safe for widespread application in food products 2011 [67]. This polymer has proven to be very versatile, with numerous applications in the food industry. It can be used to enhance the quality of food, as a film on food products, as packaging coatings, and as bioactive compounds, among other uses [66]. However, limited information is available regarding the resistance that microorganisms may develop. In this study, one strain exhibited resistance to this polymer, and additional analysis will be required to elucidate this mechanism.

### 4.7. Listeria monocytogenes Causes Infection in Chicken Embryos

Multiple eukaryotic models have been employed to evaluate the virulence of *Listeria monocytogenes.* One of these is the use of cell lines, which can be challenging to utilize in replicating the complexity of an in vivo infection [32]. Other models, like guinea pigs, gerbils, and rhesus monkeys, are hard to keep because they need special facilities. Another challenge is the high doses needed to cause infection. Models such as *Galleria mellonella*, *Drosophila melanogaster*, *Caenorhabditis elegans*, and zebrafish (*Danio rerio*) have a main limitation: their maximum development temperature is 30 °C, which is 7 degrees lower than that of humans, at 37 °C. This temperature is desired for the activation of virulence gene expression in *Listeria monocytogenes* [32].

The chicken embryo assay is a reliable, viable, and well-described method for evaluating bacterial infection in a eucaryotic model [32]. Similar survival patterns were observed when comparing the infectivity of genetically different *L. monocytogenes* strains in chicken embryos and mice. Specifically, strains that exhibited lower infectivity in the chicken embryo model also showed reduced infectivity in the mouse model.

*L. monocytogenes* wild-type isolates showed differences in the virulence pattern. Strains from fresh pork loin were less aggressive than those from packaged pork loin, and most killed the embryos at 48 h. Unlike fresh pork loin, *Listeria* strains from packaged pork loin were under cold stress. It is documented that the SigB protein can mediate the survival of *L. monocytogenes* under a wide range of lethal stresses along the food production chain, including low-temperature stress [74]. As part of the operon sigma B (*rsb* genes), these proteins can sense environmental stress conditions and regulate the SigB signaling pathway. Under adaptive stress conditions, the SigB protein can activate the PrfA transcription factor, activating virulence factors [74]. This cold stress could trigger the aggressiveness of *L. monocytogenes* isolates from freshly packaged pork loin, killing the embryos in the first 48 h, as observed previously [75,76].

Regarding the virulence factors, these results show that the absence of *actA* and *plcA* genes is not a determinant for virulence behavior in the eukaryotic model. However, the absence of *InlA*, *InlB*, *InlJ* may lead to an attenuation of virulence. It has been shown that these proteins are necessary for cellular internalization in the host. As previously demonstrated through mutation assays targeting the internalin A gene, the internalization pathway can be affected, leading to an attenuation of virulence, although it does not completely inhibit it [77]. Possibly, this infection decrease is caused by the attenuated virulence. The virulence shown by *L. monocytogenes* wild-type isolates suggests that these isolates can effectively evade cellular barriers and reach the liver in chicken embryos, demonstrating their pathogenic nature and potential risk to people who may come in contact with this strain through contaminated foods [76].

In the case of *Listeria monocytogenes* ATCC 15313, was unable to infect the chicken embryos nor infect a murine model [78]. *Listeria monocytogenes* ATCC 15313 is an avirulent strain that does not produce listeriolysin O (LLO), a well-established main virulence factor of *L. monocytogenes* [79,80], and the *hlyA* gene, which encodes this factor, is absent in this strain, resulting in its avirulent behavior [78]. The *hlyA* gene was used in PCR to detect the translocation on chicken embryos post-infection as the main virulence factor; all *L. monocytogenes* strains were detected except *Listeria innocua* ATCC 33091 and *L. monocytogenes* ATCC 15313 in liver from chicken embryos.

The results indicated variations in virulence among different isolates, with those derived from packaged pork loin showing greater aggressiveness and faster mortality rates in embryos. This study underscores the significance of considering how cold stress affects the virulence of *L. monocytogenes* in food products. It also emphasizes the need for strong food safety measures, especially for refrigerated and processed foods.

## 5. Conclusions

All the isolates obtained resulted in Listeria monocytogenes. In this study, the serotypes 1/2a and 1/2b were the most predominant in pork meat samples. A notable finding is that the prevalence varied across different sample types. The isolates analyzed showed differences in the presence of virulence factors, and we hypothesized that this could affect their pathogenic nature. Some isolates lacked certain genes (*actA*, *InlA*, *InlB*, *InlJ*, *plcA*) involved in the infection process. However, the absence of some main genes does not affect the pathogenic behavior in the eukaryotic model, except for the isolate Lm 1.2.

The antibiotic resistance shown by the microorganisms studied here is a real concern. One isolate was found to be resistant to as many as ten antibiotics, which poses a latent risk, especially considering these bacteria were isolated from food and are in close contact with people.

Chitosan has been used as a decontamination strategy, but surprisingly, one isolate exhibited resistance to chitosan. This isolate (isolate 1.2) will require further study to define its behavior regarding chitosan resistance. This could provide insight into the evolution of *L. monocytogenes* and highlight the need for caution in using chitosan as an antimicrobial compound, particularly when applied indiscriminately. Chitosan is utilized in pork production to enhance food quality and safety, serving as a natural antimicrobial agent in feed or as a protective coating for meat, thereby prolonging its shelf life.

The isolates evaluated in this work were successfully used to infect chicken embryos, resulting in death. One isolate (Lm 1.2) showed attenuated virulence; this isolate could not cause death in chicken embryos. Being the unique isolate that lacks three internalin genes, *InlA*, *InlB*, and *InlJ*, more investigations are needed to prove this.

## Figures and Tables

**Table 1:** Prevalence and frequency of *L. monocytogenes* serotypes in raw pork loin and packaged raw pork loin.

				Serotypes			
Source	Samples	Prevalence	*L. monocytogenes* Isolates Obtained	1/2a	1/2b	4b	ND
Raw pork loin	16	56.3%	9	1 (11.1%)	7 (77.8%)	–––	1 (11.1%)
Raw packaged pork loin	10	70%	7	1 (14.3%)	4 (57.1%)	1 (14.3%)	1 (14.3%)
TOTAL	26	61.5%	16	2 (12.5%)	11 (68.75%)	1 (6.25%)	2 (12.5%)

ND means serotype not identified.

**Table 2:** Virulence profiles and their respective serotypes of *L. monocytogenes* isolates from fresh pork loin and packaged fresh pork loin.

Source	Bacterial Isolate	Virulence Factors										
		*actA*	*hlyA*	*lnlA*	*InlB*	*InlC*	*InlJ*	*Iap*	*plcA*	*plcB*	*prfA*	Serotype
Raw pork loin	*L. monocytogenes* ATCC 7644 *	+	+	+	+	+	+	+	+	+	+	1/2a
	Lm 1.1	+	+	+	+	+	+	+	+	+	+	1/2b
	Lm 1.2	+	+	ND	ND	+	ND	+	+	+	+	1/2b
	Lm 1.3	+	+	+	+	+	+	+	+	+	+	1/2b
	Lm 7.1	+	+	+	+	+	+	+	+	+	+	1/2b
	Lm 7.2	+	+	+	+	+	+	+	ND	+	+	1/2b
	Lm 7.3	+	+	+	+	+	+	+	+	+	+	1/2b
	Lm Z44	+	+	+	+	+	+	+	+	+	+	1/2a
	Lm 10.1	+	+	+	ND	+	+	+	+	+	+	1/2b
	Lm 10.2	+	+	+	+	+	+	+	+	+	+	ND
Raw packaged pork loin	Lm 2.1	+	+	+	+	+	+	+	+	+	+	1/2a
	Lm 2.3	+	+	+	+	+	+	+	+	+	+	1/2b
	Lm 3.1	ND	+	+	+	+	+	+	ND	+	+	4b
	Lm 3.2	ND	+	+	+	+	+	+	ND	+	+	1/2b
	Lm 3.3	+	+	+	+	+	+	+	+	+	+	1/2b
	Lm 42.1	+	+	+	+	+	+	+	+	+	+	ND
	Lm 42.2	+	+	+	+	+	+	+	+	+	+	1/2b

* *L. monocytogenes* ATCC 7644 was used as a positive control in PCR assays. ND signifies not detected.

**Table 3:** Antibiotic compounds were evaluated against *Listeria monocytogenes* isolates from pork loin samples.

Source	Isolates	Antibiotic (MIC μg/mL) *																					
		AMC	SAM	AMP	CRO	CIP	CLI	DAP	ERY	FOF	GEN	LVX	LZD	MXF	NIT	OXA	PEN	RIF	SYN	TET	SXT	VAN	MARI
*L. monocytogenes*	ATCC ^♦^	S < 4/2	S < 8/4	S < 2	R > 32	R > 2	R > 4	R > 4	S < 0.5	S	S < 4	S < 1	S < 1	S < 0.5	S <32	R >2	S < 0.03	S <1	S < 0.5	S < 4	S < 0.5/9.5	S < 0.25	0.238
Fresh pork loin	1.1	S < 4/2	S < 8/4	S < 2	R > 32	S < 1	R > 4	R > 4	S < 0.5	S	S < 4	R > 4	R > 4	S < 0.5	R > 64	R >2	S < 0.03	S <1	S < 0.5	S <4	S < 0.5/9.5	S < 0.25	0.333
	1.2	S < 4/2	S < 8/4	S < 2	R > 32	R > 2	R > 4	R > 4	S < 0.5	S	S < 4	R > 4	R > 4	S < 0.5	R > 64	R >2	R > 8	S < 1	R > 2	S < 4	S < 0.5/9.5	S < 0.25	0.476
	1.3	S < 4/2	S < 8/4	S < 2	R > 32	S < 1	R > 4	R > 4	S < 0.5	S	S < 4	S < 1	S < 1	S < 0.5	R > 64	R >2	S < 0.03	S < 1	S < 0.5	S < 4	S < 0.5/9.5	S < 0.25	0.238
	7.1	S < 4/2	S < 8/4	S < 2	R > 32	S < 1	R > 4	R > 4	S < 0.5	S	S < 4	S < 1	S < 1	S < 0.5	R > 64	R >2	S < 0.03	S < 1	S < 0.5	S < 4	S < 0.5/9.5	S < 0.25	0.238
	7.2	S < 4/2	S < 8/4	S < 2	R > 32	S < 1	R > 4	R > 4	S < 0.5	S	S < 4	S < 1	S < 1	S < 0.5	R > 64	R >2	S < 0.03	S <1	S < 0.5	S < 4	S < 0.5/9.5	S < 0.25	0.238
	7.3	S < 4/2	S < 8/4	S < 2	R > 32	S < 1	R > 4	R > 4	S < 0.5	S	S < 4	S < 1	S < 1	S < 0.5	R > 64	R >2	S < 0.03	S < 1	S < 0.5	S < 4	S < 0.5/9.5	S < 0.25	0.238
	z44	S < 4/2	S < 8/4	S < 2	R > 32	R > 2	R > 4	R > 4	S < 0.5	S	S < 4	R > 4	S < 1	S < 0.5	R > 64	R >2	S < 0.03	S < 1	S < 0.5	R >8	S < 0.5/9.5	S < 0.25	0.381
	10.1	S < 4/2	S < 8/4	S < 2	R > 32	R > 2	R > 4	R > 4	S < 0.5	S	S < 4	S < 1	S < 1	S < 0.5	S <32	R >2	S < 0.03	S <1	S < 0.5	S <4	S < 0.5/9.5	S < 0.25	0.238
	10.2	S < 4/2	S < 8/4	S < 2	R > 32	S < 1	R > 4	R > 4	S < 0.5	S	S < 4	S < 1	S < 1	S < 0.5	R > 64	R >2	S < 0.03	S < 1	S < 0.5	S < 4	S < 0.5/9.5	S < 0.25	0.238
Fresh packaged pork loin	2.1	S < 4/2	S < 8/4	S < 2	R > 32	R > 2	R > 4	R > 4	S < 0.5	S	S < 4	S < 1	R > 4	S < 0.5	R > 64	R >2	R > 8	S < 1	R > 2	S < 4	S < 0.5/9.5	S < 0.25	0.429
	2.3	S < 4/2	S < 8/4	S < 2	R > 32	R > 2	R > 4	R > 4	S < 0.5	S	S < 4	S < 1	S < 1	S < 0.5	R > 64	R >2	S < 0.03	S < 1	S < 0.5	S < 4	S < 0.5/9.5	S < 0.25	0.286
	3.1	S < 4/2	S < 8/4	S < 2	R > 32	S < 1	R > 4	R > 4	S < 0.5	S	S < 4	S < 1	S < 1	S < 0.5	R > 64	R >2	S < 0.03	S <1	S < 0.5	S < 4	S < 0.5/9.5	S < 0.25	0.238
	3.2	S < 4/2	S < 8/4	S < 2	R > 32	R > 2	R > 4	R > 4	S < 0.5	S	S < 4	S < 1	S < 1	S < 0.5	R > 64	R >2	S < 0.03	S < 1	S < 0.5	S < 4	S < 0.5/9.5	S < 0.25	0.286
	3.3	S < 4/2	S < 8/4	S < 2	R > 32	R > 2	R > 4	R > 4	S < 0.5	S	S < 4	S < 1	S < 1	S < 0.5	R > 64	R >2	S < 0.03	S < 1	R > 2	S < 4	S < 0.5/9.5	S < 0.25	0.333
	42.1	S < 4/2	S < 8/4	S < 2	R > 32	S < 1	R > 4	R > 4	S < 0.5	S	S < 4	S < 1	S < 1	S < 0.5	R > 64	R >2	S < 0.03	S < 1	S < 0.5	S < 4	S < 0.5/9.5	S < 0.25	0.238
	42.2	S < 4/2	S < 8/4	S < 2	R > 32	R > 2	R > 4	R > 4	S < 0.5	S	S < 4	S < 1	S < 1	S < 0.5	R > 64	R >2	S < 0.03	S < 1	S < 0.5	S < 4	S < 0.5/9.5	S < 0.25	0.286

* 33 Microscan GP systems; MIC, minimum inhibitory concentration according to CLSI criteria. AMC, amoxicillin-clavulanic acid; SAM, ampicillin-sulbactam; AMP, ampicillin; CRO, ceftriaxone; CIP, ciprofloxacin; CLI, clindamycin; DAP, daptomycin; ERY, erythromycin; FOF, fosfomycin; GEN, gentamicin; LVX, levofloxacin; LZD, linezolid; MXF, moxifloxacin; NIT, nitrofurantoin; OXA, oxacillin; PEN, penicillin; RIF, rifampin; SYN, synercid; TET, tetracycline; SXT, trimethoprim-sulfamethoxazole; VAN, vancomycin. R indicates resistance and S indicates sensitivity. MARI, multiple antibiotic resistance index. ♦ *Listeria monocytogenes* ATCC 7644.

**Table 4:** Antimicrobial effect of shrimp chitosan against *L. monocytogenes* isolates from raw and packaged pork loin.

		Log (CFU/mL)			
	Isolate	Initial Inoculum	Control ^†^	Chitosan (0.25%) ^†^	Acetic Acid (1%) ^†^
Raw pork loin	Lm 1.1	6.36 ^a^	6.83 ^a^	0.00 ^b^	6.54 ^a^
	Lm 1.2	6.55 ^a^	6.76 ^a^	0.00 ^b^	6.13 ^a^
	Lm 1.3	6.18 ^a^	6.35 ^a^	0.00 ^b^	6.27 ^a^
	Lm 7.1	6.33 ^a^	6.70 ^a^	0.00 ^b^	6.12 ^a^
	Lm 7.2	6.09 ^a^	6.59 ^a^	0.00 ^b^	6.48 ^a^
	Lm 7.3	6.32 ^a^	6.71 ^a^	0.00 ^b^	6.21 ^a^
	Lm z44	6.49 ^a^	6.58 ^a^	0.00 ^b^	6.58 ^a^
	Lm 10.1	5.87 ^a^	6.58 ^a^	4.03 ^a^	6.50 ^a^
	Lm 10.2	6.39 ^a^	6.61 ^a^	0.00 ^b^	6.33 ^a^
Packaged pork loin	Lm 2.1	5.11 ^a^	5.18 ^a^	0.00 ^b^	5.11 ^a^
	Lm 2.3	5.13 ^a^	5.43 ^a^	0.00 ^b^	5.28 ^a^
	Lm 3.1	5.36 ^a^	5.53 ^a^	0.00 ^b^	5.27 ^a^
	Lm 3.2	5.55 ^a^	5.77 ^a^	0.00 ^b^	5.57 ^a^
	Lm 3.3	5.17 ^a^	5.24 ^a^	0.00 ^b^	5.25 ^a^
	Lm 42.1	5.61 ^a^	5.75 ^a^	0.00 ^b^	5.40 ^a^
	Lm 42.2	5.26 ^a^	5.63 ^a^	0.00 ^b^	5.49 ^a^

^†^ Viable cells count (Log CFU/mL) after 45min treated with chitosan (0.25%), without chitosan (control), and acetic acid (1%). Results are expressed as means. Superscript letters indicate significant differences among the samples at the same line between treatments (*p* < 0.05).

## Data Availability

The original contributions presented in the study are included in the article/Supplementary Materials, further inquiries can be directed to the corresponding author.

## Supplementary Materials

The following supporting information can be downloaded at https://www.mdpi.com/article/10.3390/foods14173057/s1: Figure S1: Evaluation of chitosan at different exposure times and chitosan concentrations on *Listeria monocytogenes* ATCC 15313; Table S1: Oligonucleotides were used for molecular analysis; Table S2. PCR detection of *Listeria monocytogenes* on chicken embryos in the virulence assay.
