# Supplementary material for: Characterization of Listeria monocytogenes Isolates from Pork Production in Southern Sonora, Mexico: Serotyping, Antimicrobial Resistance, Chitosan Susceptibility, and Pathogenicity in a Chicken Embryo Model
Source: Foods. 2025 Aug 29;14(17):3057. doi: 10.3390/foods14173057 (PMC12427709; doi:10.3390/foods14173057)
Supplement: Supplementary file 1 [file foods-14-03057-s001.zip › Figure S1.pdf]

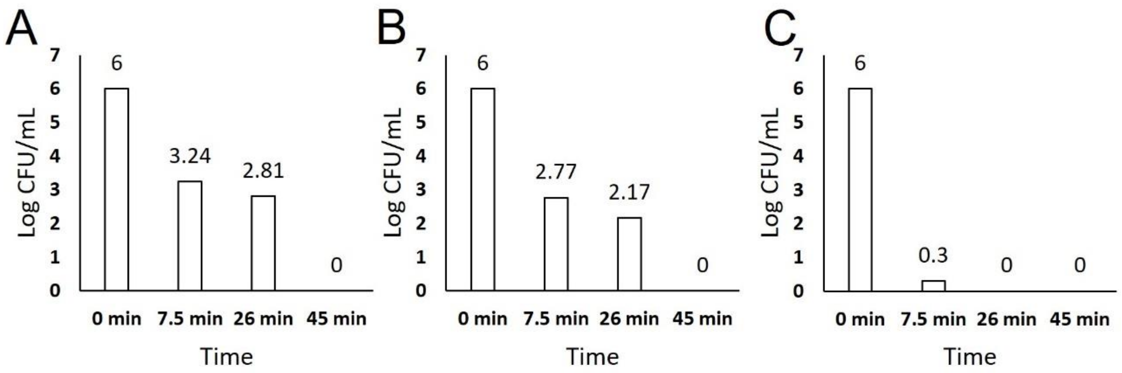

Figure S1. Evaluation of chitosan at different exposure times and concentrations on *Listeria monocytogenes* ATCC 15313. A, concentration of 0.1% of chitosan. B, 0.175% of chitosan. C, 0.25% of chitosan. The values are expressed in Log of CFU per mL.
