# Supplementary material for: Characterization of Listeria monocytogenes Isolates from Pork Production in Southern Sonora, Mexico: Serotyping, Antimicrobial Resistance, Chitosan Susceptibility, and Pathogenicity in a Chicken Embryo Model
Source: Foods. 2025 Aug 29;14(17):3057. doi: 10.3390/foods14173057 (PMC12427709; doi:10.3390/foods14173057)
Supplement: Supplementary file 1 [file foods-14-03057-s001.zip › Table S1.pdf]

Table S1 . Oligonucleotides were used for molecular analysis.

| Primer name | | Sequence (5'-3') | Size (bp) | Melting (°C) | Gen target/serotype | Reference |
|---|---|---|---|---|---|---|
| hlyA | F | gcagttgcaagcgcttggagtgaa | 456 | 56 | Gen *hlyA* | Momtaz *et al.*[13] |
| | R | gcaacgtatcctccagagtgatcg | | | | |
| iap | F | acaagctgcacctgttgcag | 131 | 58 | Gen *iap* | |
| | R | tgacagcgtgtgtagtagca | | | | |
| actA | F | cgccgcggaaattaaaaaaaga | 839 | 56 | Gen *actA* | |
| | R | acgaaggaaccgggctgctag | | | | |
| prfA | F | ctgttggagctcttcttggtgaagcaatcg | 1060 | 56 | Gen *prfA* | |
| | R | agcaacctcggtaccatatactaactc | | | | |
| plcA | F | ctgcttgagcgttcatgtctccatccccc | 1484 | 60 | Gen *plcA* | |
| | R | catgggtttcactctccttctac | | | | |
| plcB | F | ctgcttgagcgttcatgtctccatccccc | 260 | 56 | Gen *plcB* | This study |
| | R | attttcgggtagtccgcttt | | | | |
| InlA | F | accaaaaaacgccaacaaaag | 217 | 58 | Gen i*nlA* | |
| | R | atttgcggaaggtggtgtag | | | | |
| InlB | F | gttacggcaaaccagaagga | 181 | 58 | Gen i*nlB* | |
| | R | tattgtcctgctccccaaac | | | | |
| InlC | F | tgggagagtaacccaaccac | 884 | 56 | Gen i*nlC* | |
| | R | cttgaccttcgatggttgct | | | | |
| InlJ | F | gagacggcggtgtatacgat | 157 | 58 | Gen i*nlJ* | |
| | R | tctcctttgatgggttgagg | | | | |
| lmo0737 | F | agggcttcaaggacttaccc | 691 | 57 | 1/2a | Momtaz et al.[13] |
| | R | acgatttctgcttgccattc | | | | |
| ORF2819 | F | agcaaaatgccaaaactcgt | 471 | 57 | 1/2b | |
| | R | catcactaaagcctcccattg | | | | |
| ORF2110 | F | agtggacaattgattggtgaa | 597 | 57 | 4b | |
| | R | catccatcccttactttggac | | | | |
