# Supplementary material for: Characterization of Listeria monocytogenes Isolates from Pork Production in Southern Sonora, Mexico: Serotyping, Antimicrobial Resistance, Chitosan Susceptibility, and Pathogenicity in a Chicken Embryo Model
Source: Foods. 2025 Aug 29;14(17):3057. doi: 10.3390/foods14173057 (PMC12427709; doi:10.3390/foods14173057)
Supplement: Supplementary file 1 [file foods-14-03057-s001.zip › Table S2.pdf]

Table S2. *Listeria monocytogenes* PCR detection on chicken embryos in the virulence assay.

Source	Treatment	PCR detection of *hly*A gene
	Control (NaCl 0.9 %)	-
	Listeria innocua ATCC 33091	-
	Listeria monocytogenes ATCC 15313	-
	Listeria monocytogenes ATCC 7644	+
Raw pork loin	Lm 1.1	+
	Lm 1.2	+
	Lm 1.3	+
	Lm 7.1	+
	Lm 7.2	+
	Lm 7.3	+
	Lm Z44	+
	Lm 10.1	+
	Lm 10.2	+
Packaged pork loin	Lm 2.1	+
	Lm 2.3	+
	Lm 3.1	+
	Lm 3.2	+
	Lm 3.3	+
	Lm 42.1	+
	Lm 42.2	+
